# Identification of low frequency and rare variants for hypertension using sparse-data methods

**DOI:** 10.1186/s12919-016-0061-6

**Published:** 2016-10-11

**Authors:** Ji-Hyung Shin, Ruiyang Yi, Shelley B. Bull

**Affiliations:** Lunenfeld-Tanenbaum Research Institute, Sinai Health System, University of Toronto, Toronto, ON M5T 3L9 Canada

## Abstract

Availability of genomic sequence data provides opportunities to study the role of low-frequency and rare variants in the etiology of complex disease. In this study, we conduct association analyses of hypertension status in the cohort of 1943 unrelated Mexican Americans provided by Genetic Analysis Workshop 19, focusing on exonic variants in *MAP4* on chromosome 3. Our primary interest is to compare the performance of standard and sparse-data approaches for single-variant tests and variant-collapsing tests for sets of rare and low-frequency variants. We analyze both the real and the simulated phenotypes.

## Background

Despite the success of genome-wide association studies, much of the genetic contribution to complex diseases and traits remains unexplained. Therefore, an increasing number of studies have turned to low-frequency and rare variant association analysis for additional explanation of disease risk or trait variability. For binary phenotypes, single-variant analyses of low-frequency and rare variants are challenging because the conventional logistic regression approaches often violate the large-sample-size assumption for test statistics, resulting in poor type 1 error control or low statistical power [1, 2]. The standard score test, in particular, can be extremely anticonservative under the null [3]. Variant-collapsing methods across multiple variants or sparse-data methods for single-variant analysis offer an alternative [1–5]. Furthermore, depending on the linkage disequilibrium (LD) structure, it is possible that even nonfunctional low-frequency or common variants can capture functional rare variant signals [4]. On the other hand, because power is higher for a variant with a higher minor allele frequency (MAF), a common functional variant will usually be better detected by a single-variant test rather than as part of a collapsing test that incorporates nonfunctional variants.

In this report, we analyze the exome-sequence data and both the real and simulated phenotype data of the unrelated Mexican American sample to evaluate and compare the performance of single-variant and variant-collapsing methods for association analysis.

## Methods

To relate genotypes to hypertension, we consider the logistic regression model

$$ logit\left(P\left(HT{N}_i=1\ \Big| covariates\right)\right) = {\beta}_0 + AG{E}_i{\beta}_a + SE{X}_i{\beta}_s + {\boldsymbol{G}}_i{\boldsymbol{\beta}}_g, $$

where *i* = 1, …, 1943 indexes the individuals, *HTN*_*i*_ indicates hypertension status of the *i* ^*th*^ individual (1 if the individual is hypertensive and 0, otherwise); *AGE*_*i*_ is the age at the time of examination, *SEX*_*i*_ is the gender of the individual, and ${\boldsymbol{G}}_i=\left({G}_{i_1},{G}_{i_2}, \dots,\ {G}_{i_m}\right)$ indicates the vector containing the numbers of copies of the nonreference alleles at *m* variants (ie, additively coded genotype), and ${\boldsymbol{\beta}}_g\hbox{'} = \left({\beta}_1,{\beta}_2,\dots,\ {\beta}_m\right)$ is the vector of the associated parameters.

For a single-variant analysis with *m* = 1, we apply 2 types of nonstandard approaches: Firth-type penalized logistic regression likelihood ratio (LR) tests [2, 6–8], and small-sample adjusted score tests [9], and compare them to standard LR and score tests. The LR and score tests are asymptotically equivalent but may be discrepant in finite samples. The penalized LR test is based on the penalized log-likelihood function

$$ {l}_p\left(\boldsymbol{\beta} \right)=l\left(\boldsymbol{\beta} \right)+\frac{1}{2} \log \left(\left|i\left(\boldsymbol{\beta} \right)\right|\right), $$

where *i*(***β***) is the Fisher information matrix. This is a generalization of Haldane’s statistic for sparse 2 × 2 table analysis, where $\frac{1}{2}$ is added to each cell. For the small-sample-adjusted single-variant score tests, we apply an approach to adjust the null distribution of the test statistic by incorporating small-sample variance and/or kurtosis (see Lee et al. [9], pp. 226–227); this approach was originally recommended for variant-collapsing tests.

For variant-collapsing analysis, we consider a MAF-based weighted burden test [1], a nonburden sequence kernel association test (SKAT) and a unified approach (SKAT-O) that optimally combines a burden test and a SKAT (eg, Lee et al. [9]). For these tests, we first define *K* subregions, then pool the variants within each subregion, and test *K* null hypotheses ${H}_{0_K}:\left({\beta}_1,{\beta}_2,\dots,\ {\beta}_{m_k}\right)\hbox{'}=\left(0,\ 0,\dots,\ 0\right)\hbox{'}$, where *m*_*k*_ indicates the number of variants within the *k*-th subregion (*k* = 1,…, *K*). For convenience, we determine the subregions on the basis of physical proximities among the variants.

**Fig. 1:**
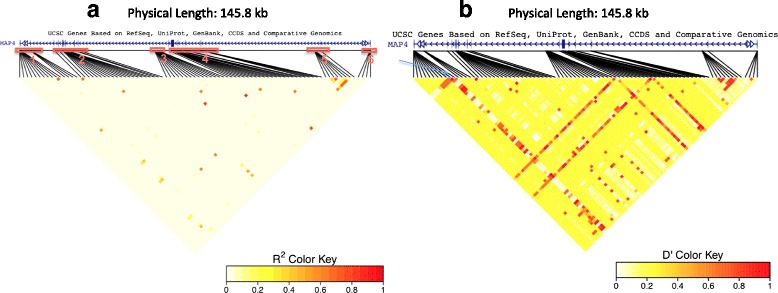
Pairwise LD measures for markers within *MAP4* region on chromosome 3 in 1943 unrelated samples. The hg19 genome assembly was used for annotation. In panel (**a**), each pixel represents pairwise LD, measured by the squared allelic correlation coefficient *r* ^2^ between 2 markers. In panel (**b**), LD is measured by Lewontin’s |*D* ^'^|. The latter are generally higher because |*D* ^'^| takes into account that the correlation is constrained by the allele frequencies. As indicated by the color key, stronger LD is represented by red and weaker by white. The LD plot was produced using the LDheatmap package [16]

Applying these methods, we analyzed exonic variants within *MAP4* gene on chromosome 3 in the real and the simulated phenotype data sets. For the imputed variants, we analyzed the predicted dosages rather than their best-guess genotypes. In addition, we examined all polymorphic variants, including the singletons to assess the extremes at which the tests break down. For the standard and penalized logistic regression tests, we used the R *glm* function and *pmlr* (Penalized Multinomial Logistic Regression) package [10], respectively. For the small-sample-adjusted score test and the variant-collapsing tests, we used the R package *SKAT* [11], with analytical variance estimates and empirical kurtosis estimates based on 10,000 bootstrap replicates. For the variant-collapsing methods, we let *K* = 6 based on a visual inspection of the physical positions of the variants (Fig. 1a).

### Data preparation

In the real data set, we defined the hypertension phenotype using the conventional diagnostic criteria: a systolic blood pressure (SBP) greater than 140 mm Hg or a diastolic blood pressure (DBP) greater than 90 mm Hg. We also defined individuals on antihypertensive medication to be hypertensive regardless of their SBP and DBP levels.

For the simulated phenotypes, 2 data sets were available, “SIMQ1” and “SIMPHEN,” each with 200 replicates. SIMQ1, designed for evaluating type 1 error rates, contained normally distributed *Q*_1_ generated under no genetic effects. Because SIMQ1 did not have binary phenotypes, we dichotomized *Q*_1_ to create hypothetical disease status *Q*_2_, letting *Q*_2_ correlate with AGE and SEX through *Q*_1_. We let *Q*_2_ = 1 if *Q*_1_ was greater than 51.2 and 0 otherwise, such that the disease prevalence for *Q*_2_ was 17.8 %, the same as the prevalence of hypertension in SIMPHEN, which we used for evaluating power. The hypertension phenotype was derived from blood pressure phenotypes generated under a model with more than 1000 variants in more than 200 genes [12].

## Results

### *MAP4* variants in the unrelated sample

**Fig. 2:**
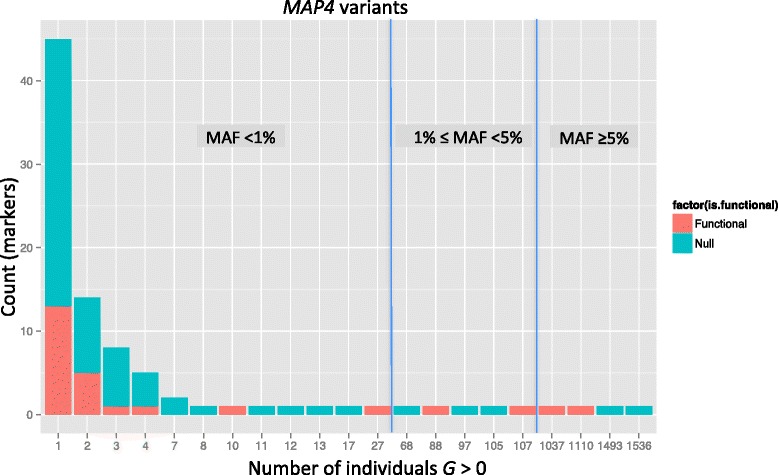
Distribution of the frequency for the 90 polymorphic *MAP4* variants according to the number of individuals with genotype dosage *G* > 0. Height of the bars indicates the total number of variants for a given count of observations with *G* > 0, and red bars indicate the counts for the 26 functional variants used in the simulation model

Of the 409 exonic *MAP4* variants, only 90 were polymorphic in the sample of 1943 unrelated individuals. These variants had MAFs ranging from 0.00027 to 0.34. As expected, rare variants (MAF <1 %) were most prevalent in the sample; except for 4 common variants, all variants had MAF less than 5 % (Fig. 2, Table 1). As expected for rare variants (eg, Pritchard [13]), the pairwise LD in the 90 variants was generally weak, with the exception of a few variants in strong LD in an upstream region (see Fig. 1). However, the strong LD seems to arise because of their physical proximities (all the markers in the LD block are located within 39 bases).

**Table 1:** Frequencies of rare (MAF **<**1 %), low-frequency (1 % **≤** MAF <5 %) and common (MAF ≥5 %) variants in *K* = 6 subregions within *MAP4* region on chromosome 3

Subregion	Variant IDs	Rare^a^	Low-frequency^a^	Common^a^	Total^a^
1	1–16	14 (4)	–	2 (0)	16 (4)
2	17–38	20 (7)	2 (0)	–	22 (7)
3	39–53	15 (0)	–	–	15 (0)
4	54–77	20 (10)	2 (1)	2 (2)	24 (13)
5	78–86	9 (0)	–	–	9 (0)
6	87–90	3 (1)	1 (1)	–	4 (2)
Total	–	81 (22)	5 (2)	4 (2)	90 (26)

^a^The values in parentheses indicate the numbers of variants designated as functional in the simulation study

### Analysis of the real phenotype data

We found that the standard score test rejects the null hypothesis far more often than the other single-variant tests (results not shown), suggesting that it may be anti-conservative. This agrees with published simulations under a case-control design [3] and is confirmed by our own unpublished simulation studies under a cohort design at the observed hypertension prevalence of 26 %. After correcting for multiple testing, no single-variant tests identified any association (minimum unadjusted *p* value = 0.006). The burden, SKAT and SKAT-O (optimal sequence kernel association test) tests, each of which pooled all polymorphic variants within the *K* = 6 subregions defined in Table 1 and Fig. 1a, did not find the *MAP4* gene to be significant either (minimum unadjusted *p* values = 0.12, 0.24, and 0.20, respectively).

### Analysis of the simulated phenotype data

It has been demonstrated that for a genome-wide study with a large sample size, minor allele count (MAC) is the key parameter determining test calibration [3]. Because we analyzed predicted dosages, we do not have a MAC for all the variants. Hence, for the presentation of simulation results, we use the count of individuals with *G* > 0 dosage, denoted by $\tilde{MAC}$, which is close to the MAC for a low MAF. For type 1 error rates of the single-variant tests, we pooled the results across all variants with the same values of $\tilde{MAC}$. Power for the single-variant tests was evaluated separately for each of the 26 functional variants. For the variant-collapsing tests, power was examined for each subregion containing at least one functional variant.

#### Test size and type I error

**Fig. 3:**
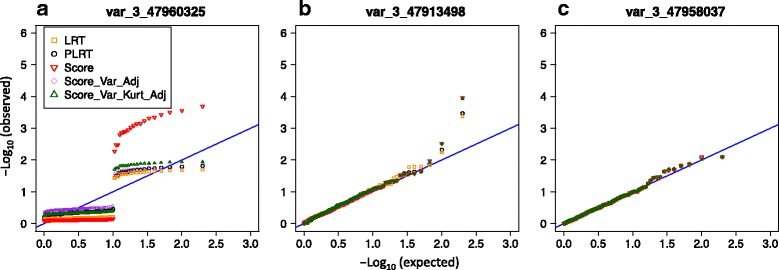
Q-Q plots of *p* values from the single-variant tests under the null hypothesis. The *p* values from the standard likelihood ratio test (LRT), penalized likelihood ratio test (PLRT), standard score test (Score) and small-sample-adjusted score tests (Score-Var-Adj and Score-Var-Kurt-Adj) are indicated by yellow squares, black circles, red point-down triangles, purple diamonds, and green point-up triangles, respectively. Panels (**a**), (**b**), and (**c**) show a rare, a low-frequency, and a common variant with $\tilde{MAC}$ = 1, 87, and 1065 observations with genotype dosage *G* > 0. The results are based on 200 replicates of the null binary phenotypes *Q*_2_

Examination of quantile–quantile (Q-Q) plots of the single-variant test *p* values for rare variants revealed departures from the expected distribution under the null hypothesis of no genetic effects with some discrepancies among tests. For example, for var_3_47660325, with $\tilde{MAC}=1,$ all the single-variant tests showed unusual departures from the expected (Fig. 3a). For low-frequency variants, the *p* value distributions were close to the expected, except in the upper tail where all tests seemed to be anticonservative (eg, Fig. 3b). As expected, the common variant test *p* values were close to the null distribution with no discrepancy among the tests (eg, Fig. 3c).

**Fig. 4:**
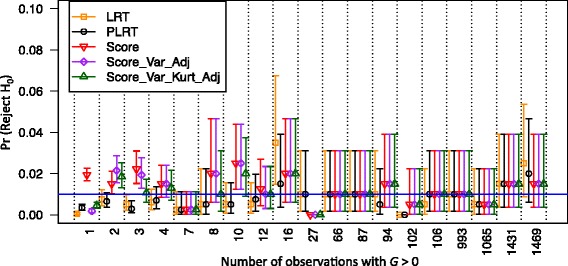
Empirical type 1 error rates of the single-variant tests at significance level of 0.01, according to the number $\tilde{MAC}$ of observations with genotype dosage *G* > 0 in the 1862 individuals with complete information on AGE and SEX. For the count-specific assessment, the results were pooled for the variants with the same $\tilde{MAC}$ value, and the proportions of the *p* values <0.01 were then computed. Tests are the standard likelihood ratio test (LRT), penalized likelihood ratio test (PLRT), standard score test (Score) and small-sample-adjusted score tests (Score-Var-Adj and Score-Var-Kurt-Adj), which are indicated by yellow squares, black circles, red point-down triangles, purple diamonds, and green point-up triangles, respectively. The vertical line segments indicate ±2 simulation error bars, which were calculated based on the total number of the polymorphic variants in each $\tilde{MAC}$ -specific group. For example, for the variants with $\tilde{MAC}$ = 1, the error bars were obtained based on 200 × 44 = 8800 simulated data sets

Examination of empirical type 1 error rates for the single-variant tests demonstrates that no method performed uniformly better than others for the rare variants with very low MACs (Fig. 4). For example, when $\tilde{MAC}$ is less than 15, the standard score test tended to be anticonservative at a significance testing level of 0.01 (Fig. 4), but was conservative at the less stringent significance level of 0.05 (results not shown). The standard LR test tended to be conservative for low $\tilde{MAC}$ (eg, <10), but could be anticonservative when this count was between 10 and 20. Although the 2 small-sample score tests could also be anticonservative, and the penalized LR test tended to be conservative in general, the type 1 error rates of these tests were closer to the nominal level than the standard tests. When $\tilde{MAC}$ is 66 or greater (or MAF >1 %), all the single-variant tests seem to control type 1 error reasonably well.

#### Power

**Fig. 5:**
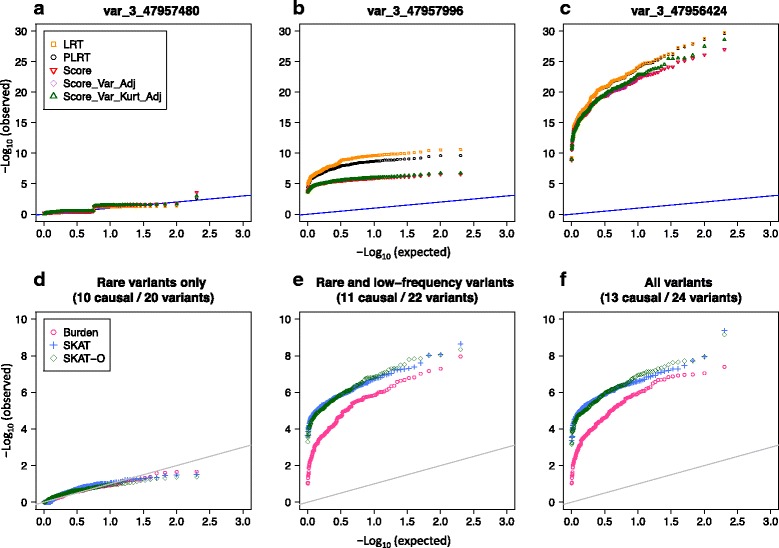
Q-Q plots of the *p* values from the single-variant tests and variant-collapsing tests of markers in subregion 4 (see Table 1 and Fig. 2a). Panels (**a**) to (**c**) show the *p* values from the single-variant tests for a rare, low-frequency, and common functional variant, respectively; the tests are the standard likelihood ratio test (LRT), penalized likelihood ratio test (PLRT), standard score test (Score) and small-sample-adjusted score tests (Score-Var-Adj and Score-Var-Kurt-Adj), which are indicated by yellow squares, black circles, red point-down triangles, purple diamonds, and green point-up triangles, respectively. Panels (**d**) to (**f**) show the results from the variant-collapsing tests when they include the rare variants, rare and low-frequency variants, and all the variants within the region. The *p* values from the weighted burden tests, SKAT, and SKAT-O are, respectively, represented by pink circles, blue cross marks, and green diamonds. The calculations are based on 200 simulated data sets in SIMPHEN

All the single-variant tests had power of less than 20 % to detect *each of* the rare variants, but had 100 % power for the low-frequency and the common variants at the significance levels of 0.01 and 0.05. For the low-frequency variants, tests had discrepant *p* values, and differential power at a stricter significance level. For example, in Fig. 5b, all the tests for var_3_47957996 (MAF = 0.0024) had *p* values of less than 0.01; however, the 2 LR tests had consistently lower *p* values than the 3 score tests. At a significance level of 1e–06, the standard and penalized LR tests had 91 and 82 % power, respectively, whereas the standard, the small-sample-variance, and the small-sample-variance-kurtosis score tests had less than 10 % power.

Among the 4 subregions with at least 1 functional variant, power was nonnegligible only in subregions 4 and 6 (Table 2). Figure 5d-f shows the results from the variant-collapsing tests of the markers in subregion 4, which contains all 3 types of functional variants (rare, low-frequency, and common). As expected, the burden test tended to have lower power than SKAT or SKAT-O because the subregion includes both protective and deleterious variants. These tests all had low power when the subregion includes only rare variants (eg, Fig. 5d). The power improved when the subregion included both the rare and the low-frequency functional variants (eg, Fig. 5e). When, however, the common variants were additionally included, the power did not seem to improve further (Fig. 5f). When compared with the single-variant tests of markers in the same subregion (Fig. 5b and c), the results suggest that this subregion would have been detected by some of the single-variant tests, as well, even at the genome-wide significance level of 5e–08.

**Table 2:** Empirical power estimates of the collapsing-variant tests based on 200 simulated data sets in SIMPHEN, according to the *MAP4* subregions containing at least 1 functional variant

		Burden^a^			SKAT^a^			SKAT-O^a^		
Significance level	Subregion	Rare	Rare & low- frequency	All	Rare	Rare & low- frequency	All	Rare	Rare & low- frequency	All
0.01	1	0	0	0.260^b^	0.005	0.005	0.840^b^	0.005	0.005	0.805^b^
	2	0	0.005	0.005	0	0.145	0.145	0	0.090	0.080
	4	0	0.955	0.955	0	1.000	1.000	0	1.000	1.000
	6	0	0.990	0.995	0	1.000	1.000	0	1.000	1.000
0.05	1	0.045	0.045	0.555^b^	0.015	0.020	0.960^b^	0.015	0.015	0.970^b^
	2	0.060	0.030	0.030	0.020	0.330	0.330	0.005	0.255	0.250
	4	0.030	0.990	0.990	0.025	1.000	1.000	0.015	1.000	1.000
	6	0.105	1.000	1.000	0.075	1.000	1.000	0.085	1.000	1.000

^a^For each subregion, power was estimated when a test includes only rare, rare and low-frequency, and all variants

^b^A result of 2 *nonfunctional* common variants that were in LD (*r* ^2^ > 0.3) with the 2 functional common variants in subregion 4

## Discussion and conclusions

In this article, we evaluated standard and sparse-data methods for single-variant and variant-collapsing tests to examine the association between a hypertension phenotype and exonic variants in *MAP4* gene on chromosome 3, using both the real and the simulated phenotypes in unrelated Mexican Americans.

In the analysis of the real phenotype data, none of the single-variant and the variant-collapsing methods detected *MAP4* variants significantly associated with hypertension. A limitation of our analysis is that we did not make any adjustment for ancestry admixture/population structure. In genetic association studies of admixed populations such as Mexican Americans, addressing differential ancestral backgrounds is important to avoid false positive or negative association signals [14, 15].

In our simulation investigation, we found that the sparse-data approaches improve type 1 error control, but their power remains low for detecting the rare variant effects. Because power of the association tests depends on both frequency and effect size of rare variants, even with large effects, the tests may detect rare variants only in studies with large samples. We may be more successful in identifying rare variants when we use joint or meta-analyses combining data or summary statistics from different studies (eg, Ma et al. [3]). For the low-frequency variants, all the single-variant tests seem to have improved type 1 error rates and power. It seems that the LR tests have higher power than the score tests at a stringent significance level. However, we cannot make any concrete conclusions because of the limited number of replications provided in the simulation design. Although more thorough investigation is necessary, overall, the penalized LR test and the score test with small-sample variance and kurtosis seem to be better choices than the standard tests for the analyses of rare and low-frequency variants. Moreover, caution is indicated when different tests of the same hypothesis give inconsistent *p* values as it suggests large-sample approximations for test statistics may be invalid.

Although previous simulation studies have shown that collapsing tests can have greater power than single-variant tests (see, eg, Madsen and Browning [1]), our investigation suggests that power of collapsing tests can be low when the tests include only the rare variants (see, eg, Fig. 5d). In addition to MAF and effect size, power of collapsing tests depends on the number of associated variants, the number of neutral variants, and whether the direction of effects is consistent within gene, so that selection of good binning and weighting strategies may boost power for detecting regions containing only rare variants.

## References

[1] Madsen BE, Browning SR (2009). A groupwise association test for rare mutations using a weighted sum statistic. PLoS Genet.

[2] Heinze G, Schemper M (2002). A solution to the problem of separation in logistic regression. Stat Med.

[3] Ma C, Blackwell T, Boehnke M, Scott LJ, the GoT2D investigators (2013). Recommended joint and meta-analysis strategies for case-control association testing of single low-count variants. Genet Epidemiol.

[4] Kinnamon DD, Hershberger RE, Martin ER (2012). Reconsidering association testing methods using single-variant test statistics as alternatives to pooling tests for sequence data with rare variants. PLoS One.

[5] Kosmidis I (2014). Bias in parametric estimation: reduction and useful side-effects. Wiley Interdiscip Rev Comput Stat.

[6] Firth D (1993). Bias reduction of maximum likelihood estimates. Biometrika.

[7] Bull SB, Mak C, Greenwood CM (2002). A modified score function estimator for multinomial logistic regression in small samples. Comput Stat Data Anal.

[8] Bull SB, Lewinger JP, Lee SS (2007). Confidence intervals for multinomial logistic regression in sparse data. Stat Med.

[9] Lee S, Emond MJ, Bamshad MJ, Barnes KC, Rieder MJ, Nickerson DA, Christiani DC, Wurfel MM, Lin X (2012). Optimal unified approach for rare-variant association testing with application to small-sample case-control whole-exome sequencing studies. Am J Hum Genet.

[10] Colby S, Lee S, Lewinger PJ, Bull SB: pmlr: Penalized Multinomial Logistic Regression. R package version 1.0; 2010. http://CRAN.R-project.org/package=pmlr.

[11] Lee S, Miropolsky L, Wu M: SKAT: SNP-Set (Sequence) Kernel Association Test. R package version 0.95; 2014. http://CRAN.R-project.org/package=SKAT.

[12] Blangero J, Teslovich TM, Sim X, Almeida MA, Jun G, Dyer TD, Johnson M, Peralta JM, Manning AK, Wood AR, et al. Omics squared: Human genomic, transcriptomic, and phenotypic data for Genetic Analysis Workshop 19. BMC Proc. 10.1186/s12919-016-0008-y PMC5133484 27980614

[13] Pritchard JK (2001). Are rare variants responsible for susceptibility to complex diseases?. Am J Hum Genet.

[14] O'Connor TD, Kiezun A, Bamshad M, Rich SS, Smith JD, Turner E, Leal SM, Akey JM, NHLBIGO Exome Sequencing Project; ESP Population Genetics, Statistical Analysis Working Group (2013). Fine-scale patterns of population stratification confound rare variant association tests. PLoS One.

[15] Bermejo JL (2015). Above and beyond state-of-the-art approaches to investigate sequence data: Summary of methods and results from the Population-based Association Group at the GAW 19. BMC Genet.

[16] Shin J-H, Blay S, McNeney B, Graham J: LDheatmap: an R function for graphical display of pairwise linkage disequilibria between single nucleotide polymorphisms. J Stat Softw. 2006;16: Code Snippet 3.

